# Enhanced classifier training to improve precision of a convolutional neural network to identify images of skin lesions

**DOI:** 10.1371/journal.pone.0218713

**Published:** 2019-06-24

**Authors:** Titus J. Brinker, Achim Hekler, Alexander H. Enk, Christof von Kalle

**Affiliations:** 1 National Center for Tumor Diseases (NCT), German Cancer Research Center (DKFZ), Heidelberg, Germany; 2 Department of Dermatology, University Hospital Heidelberg, Heidelberg, Germany; The University of Western Australia, AUSTRALIA

## Abstract

**Background:**

In recent months, multiple publications have demonstrated the use of convolutional neural networks (CNN) to classify images of skin cancer as precisely as dermatologists. However, these CNNs failed to outperform the International Symposium on Biomedical Imaging (ISBI) 2016 challenge which ranked the average precision for classification of dermoscopic melanoma images. Accordingly, the technical progress represented by these studies is limited. In addition, the available reports are impossible to reproduce, due to incomplete descriptions of training procedures and the use of proprietary image databases or non-disclosure of used images. These factors prevent the comparison of various CNN classifiers in equal terms.

**Objective:**

To demonstrate the training of an image-classifier CNN that outperforms the winner of the ISBI 2016 CNNs challenge by using open source images exclusively.

**Methods:**

A detailed description of the training procedure is reported while the used images and test sets are disclosed fully, to insure the reproducibility of our work.

**Results:**

Our CNN classifier outperforms all recent attempts to classify the original ISBI 2016 challenge test data (full set of 379 test images), with an average precision of 0.709 (vs. 0.637 of the ISBI winner) and with an area under the receiver operating curve of 0.85.

**Conclusion:**

This work illustrates the potential for improving skin cancer classification with enhanced training procedures for CNNs, while avoiding the use of costly equipment or proprietary image data.

## Introduction

Skin cancer is the most common malignancy in fair-skinned populations, and melanoma accounts for the majority of skin cancer-related deaths worldwide[1,2]. In light of the rapid increase in the prevalence of melanoma over recent decades, several institutions and entities have emphasized and funded programs to improve measures for early detection, including photoaging mobile apps [3–7] or screening of individuals in risk groups [8].

Dermoscopes significantly improve the diagnostic accuracy of naked-eye examinations when screening for skin cancer [9]. Despite special training, however, general medical practitioners and dermatologists rarely exceed sensitivity greater than 80% [10;11].

In 2017, Esteva et al. reported a deep-learning convolutional neural network (CNN) image classifier that performed as well as 21 board-certified dermatologists when identifying images with malignant lesions [12]. The CNN deconstructed digital images of skin lesions into pixel-level maps, and generated its own diagnostic criteria for melanoma detection during training. The test or training data has not been released to the public, however, and Esteva et al. did not test their classifier with a publicly available benchmark, so reproduction or validation of the results is impossible.

In recent months, several publications have demonstrated dermatologist-level skin cancer classification via deep neural networks (CNN) [12–19]. None of these CNNs proved to outperform the ISBI 2016 skin cancer classification challenge in terms of average diagnostic precision, so the reported technical progress is limited [15,20]. In addition, the first systematic review of this literature pointed to the lack of reproducibility of these studies, due to incomplete descriptions of training procedures and the use of proprietary image databases [20].

In general, the classification of images of skin cancer via a deep learning algorithm may be improved by three strategies: using a greater number of images of positive biopsies in training; increasing the capacity of the graphic processor units (GPUs), or modifying the training procedure.

In this article, we demonstrate the improved training of a CNN classifier that outperforms the winner of the ISBI 2016 challenge. The classifier was trained exclusively on dermoscopic images from the publicly available ISIC archive. The level of detail in this report ensures the reproducibility of our work.

## Methods

The ethics committee of the University of Heidelberg waived the need to obtain an ethics vote due to the fact that there are no patient or healthy test person samples involved and the used images are anonymous.

This article considers the binary classification problem of sorting images into groups of malign and benign skin lesions. We assume that the images of skin lesions have been labeled by experts, and that dermoscopic images are used as inputs for the classifier.

### CNN model and training procedures

We developed an image classifier with the convolutional neural network (CNN) architecture. CNNs have shown excellent performance for image classification in many domains [21]. Roughly, CNNs identify patterns in the raw pixels of the input image that relate to the classification labels.

In this study, a ResNet50 model was used to classify images into groups of malign vs. benign skin lesions [22]. Ensembles of such residual nets dominated the ImageNet 2015 contest and achieved nearly half the error rate of the previous winner winner GoogLeNet [23]. In our work, the ResNet50 network was pre-trained with the ImageNet dataset, which includes 1.28 million images grouped into 1000 object classes. The pre-trained model was then fine-tuned for the binary skin-lesion classification task by replacing the last layer to allow only two-dimensional outputs. The parameters of the CNN were then optimized using transfer learning.

In contrast to existing approaches for skin lesion classification [4,5] which implement transfer learning in a very generic manner, we applied three successive specific training techniques that increase the quality of the resulting classifier:

1. Exclusive training of the adapted last layer for a few epochs
2. Fine-tuning the parameters of all layers with learning rates specific to each layer
3. Sudden increases of the learning rate at specific time steps during fine-tuning

In the proposed method, the parameters of all layers except the last fully-connected layer are frozen at first; only the parameters of the last layer are trained with a learning rate of 0.01 over three epochs.

Next, all layers of the CNN are fine-tuned. Though existing approaches apply the same learning rate for all layers of the CNN, we implement different learning rates for each layer. Layers closer to the input are assigned slower learning rates, and layers further from the inputs are assigned faster learning rates. This application of differential learning rates is intended to encourage the layers that are closer to the input to recognize more-general image features such as edges or gradients. Then these layers do not need to be trained specifically to pick out patterns relevant to skin lesions. In contrast, the latter layers will register application-specific features, which is accomplished with the enhanced learning rate for these layers in the transfer-learning phase.

The layers are split into three groups with different learning rates for each group: the first 6 residual units have a learning rate of 0.009, the subsequent 8 residual blocks 0.003, and the fully connected layers 0.01. We based our selection of these three learning rates on our experience with other image-classification tasks.

In each batch of stochastic gradient descent, or in other words each step of optimization over the parameters of the network, the parameters approach the minimum value of the loss function. As the parameters approach this limit, the learning rate is commonly decreased incrementally to ensure that the optimization settles as close as possible to the minimum value of the loss function without overshooting it [12,24]. Instead of this gradual tapering of the learning rate, we implemented the so-called cosine annealing technique, which decreases the learning rate according to the cosine function [25].

During optimization of the network parameters, the gradient descent is at risk of settling into a local minimum instead of a global minimum of the loss function. The gradient descent can be disturbed by these local minima with sudden increments of the learning rate at specific time steps, which helps to ensure that the training reaches a global minimum of the loss function. This technique is called stochastic gradient descent with restarts (SGDR), and has been shown to be highly effective for improving the classification performance of CNNs over those trained with stochastic gradient descent alone [25][25].

We used the PyTorch deep learning framework to train, validate and test our network. During training, the number of images was increased by data augmentations including rotation, flipping, random crop, change of lighting, and enlargement. The dimensions of all images were forced to be 299 x 299 with cropping or padding.

### Training, validation, and test data

Dermoscopic images were taken from the public ISIC archive, and only images of melanomas and nevi were exported. The image archive includes a total of 2132 melanomas and 18170 nevi. The diagnoses of all melanomas in these images were verified via biopsy. The diagnoses of nevi were made either by histopathological examinations (~ 24%), by expert consensus (~54%), or by another type of diagnosis such as a series of images with no change over time (~22%).

For testing, we used the original test data set from the ISBI 2016 challenge consisting of 379 images of benign and malign skin lesions. To address the imbalance of the classes in the test data, images of nevi from the ISIC archive were selected randomly until the ratio between melanomas and nevi was 1:5. Then the validation set was generated by randomly selecting 10% of the images in this set. Finally, the test set and the validation set were deleted from the total set of images to ensure that the test set, validation set, and training set were disjoint. After this process, the training set consists of 1888 melanomas and 10490 nevi, the validation set contains 210 melanomas and 1049 nevi, and the test set contains 304 benign and 75 malign skin lesions.

## Results

**Fig 1:**
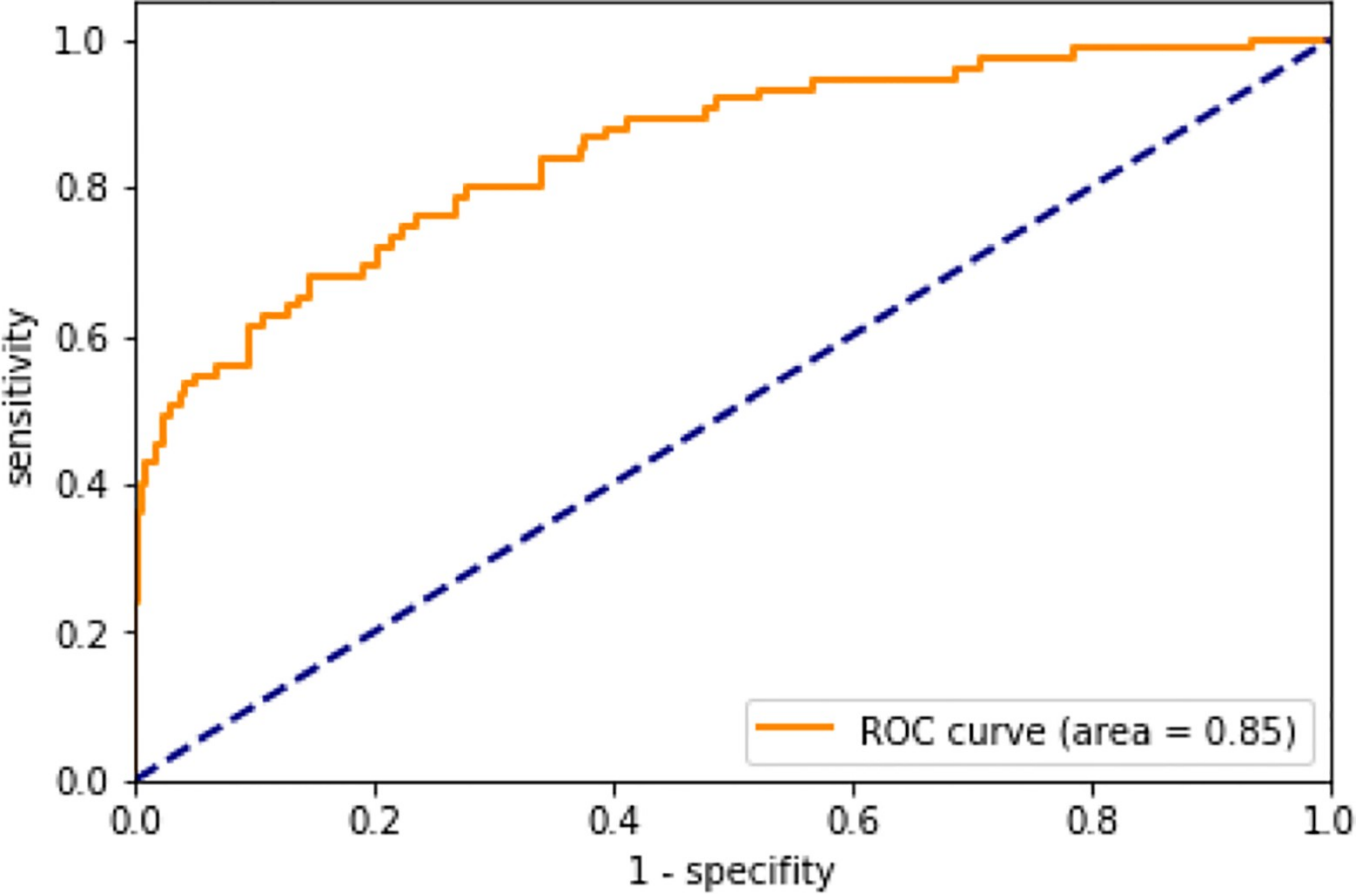
Receiver operating characteristic (ROC) curve of the proposed skin classifier tested on the ISBI 2016 challenge data.

When classifying images from the ISBI 2016 challenge dataset, our CNN classifier achieved an average precision of 0.709. This corresponds to an improvement of 11.3% over the precision of the winner of the ISBI 2016 challenge (Table 1). Other results included accuracy of 83.9%, sensitivity of 56%, specificity of 90.8%, and an area under the receiver operating curve (AUC ROC) of 0.85 were achieved. Table 1 lists the test results from our CNN, results from the participants in the ISBI 2016 challenge, and results published by Haenssle et al [15]. The receiving operating characteristics (ROC) curve for our CNN classifier is depicted in Fig 1.

**Table 1:** Results of participants of the International Symposium on Biomedical Imaging (ISBI) 2016 challenge with the result published by Haenssle et al.

	Average Precision	Accuracy	AUC ROC	Sensitivity	Specificity
**Our approach**	0.709	0.839	0.85	0.56	0.908
Rank 1	0.637	0.855	0.804	0.507	0.941
Rank 2	0.619	0.813	0.802	0.573	0.872
Rank 3	0.598	0.834	0.826	0.32	0.961
Rank 4	0.563	0.786	0.796	0.667	0.816
Rank 5	0.559	0.844	0.775	0.24	0.993
Haenssle et al.	not listed	not listed	0.79	not listed	not listed

## Discussion

The output of a classifier can be interpreted as a discrete probability distribution over the classes in question. When performing a binary classification, the output vector is two-dimensional. The first dimension of the vector quantifies the probability that the input image belongs to class A, whereas the second dimension of the vector reflects the probability that the input belongs to class B. Normally, inputs with a corresponding output value greater than 0.5 are assigned to class B and those with an output value less than 0.5 are assigned to class A. Using a threshold of 0.5, our CNN achieves a sensitivity of 56% and a specificity of 90.8%. In a clinical setting, it is of course important that skin cancer classifications have high sensitivity and the highest-possible corresponding specificity. The trade-off between sensitivity and specificity can be adjusted by varying the threshold between 0 and 1. A threshold lower than 0.5 increases the sensitivity and decreases the specificity and vice versa. The trade-off between sensitivity and specificity is illustrated by the ROC curve in Fig 1. Most of the classifiers initially developed for skin cancer classification were based on transfer learning using various CNN models such as AlexNet, GoogLenet, or VGGNet. All approaches published so far use images from the ImageNet database as inputs for pre-training. The best results so far have been achieved by fine-tuning the pre-trained parameters of all the layers in the network, while our method focused fine-tuning on layers furthest from the input.

We achieved an initial performance improvement by training only the parameters of the last layer with a few epochs before fine-tuning all the layers. In contrast to existing approaches, the fine-tuning step had differential learning rates to the layers in the CNN. The parameters of the layers closest to the input were therefore adjusted less than those of the latter layers. To quantify the improvement of these learning techniques, we conducted simulations with the same classification settings but without pre-training the last layer, and using a constant learning rate of 0.01 instead of a differential learning rate. After 12 training epochs, the average mean precision of the classifier working on the ISBI 2016 test set was 0.6015. A precision improvement of 0.1 was therefore accomplished with carefully designed training techniques alone.

Further improvements leading to the ultimate results of our approach (Table 1) were achieved by selecting the appropriate CNN model. During the evaluation of our approach, we found that the ResNet50 model was most suitable for classification of benign and malign skin lesions, when compared with, for example the ResNet34 or VVGNet model.

While new datasets and challenges have been released, the comparison to the ISBI 2016 challenge is still relevant. The paper focuses not on mere outperformance but shows that by solely using novel deep learning techniques, an outperformance of previous classifiers can be achieved. This comparison indicates a rapid development of the field of deep learning and shows that no costly computational resources or additional proprietary image data is strictly necessary for outperformance.

### Inaccuracy of the current reference standard (biopsy)

If a skin-lesion classifier is to be clinically useful, the diagnostic accuracy of the method used (i.e. a CNN) must be comparable to the current standard of care. For instance, in case a biopsy was taken, the reference standard is the result of histopathologic tests, but if the lesion is considered benign, the clinical diagnosis by the specialist is accepted as the reference standard. In addition, a 2010 study of discordance in the histopathology for melanoma diagnosis in the US reported discordant results in 392 cases, or 14.3% of the study sample [26], and a 2016 study from another US center reported an even higher discordance in 19% of 600 sample cases, indicating that the problem of discordant histopathology not improved over time [27]. Such discrepancies in the reference standard have clinical consequences for patients, and they influence studies of diagnostic accuracy [26–28]. Diagnostic discordance needs to be considered when discussing the thresholds for acceptable levels of diagnostic accuracy when developing new diagnostic measures. While biopsy-classified images remain the gold standard for accurate classifier training, they must be used at least in the test set that aims at estimating the accuracy of the CNN. They should also be used when training the classifier, in ideal circumstances. In the long run, the current gold standard needs to be made more precise, or a different method needs to be proposed.

## Conclusions

Our work illustrates how to improve the precision of CNN skin-cancer classifiers with training procedures alone—regardless of additional computational resources or additional proprietary image data. This makes the approach realistic for replication and allows for easier translation into a real-world scenario. Future research should confirm our findings in prospective studies and include a broader differential diagnosis in the algorithms’ training.
